# Informed Consent Decision-Making in Deep Brain Stimulation

**DOI:** 10.3390/brainsci8050084

**Published:** 2018-05-11

**Authors:** Gabriele Mandarelli, Germana Moretti, Massimo Pasquini, Giuseppe Nicolò, Stefano Ferracuti

**Affiliations:** 1 Department of Human Neurosciences (Former Department of Neurology and Psychiatry), “Sapienza” University of Rome, 00185 Rome, Italy; massimo.pasquini@uniroma1.it (M.P.); stefano.ferracuti@uniroma1.it (S.F.); 2 Department of Mental Health, ASL Roma 5, 00034 Colleferro, Italy; germana.moretti@aslromag.it (G.M.); giuseppe.nicolo@aslromag.it (G.N.)

**Keywords:** informed consent, decision-making capacity, deep brain stimulation

## Abstract

Deep brain stimulation (DBS) has proved useful for several movement disorders (Parkinson’s disease, essential tremor, dystonia), in which first and/or second line pharmacological treatments were inefficacious. Initial evidence of DBS efficacy exists for refractory obsessive-compulsive disorder, treatment-resistant major depressive disorder, and impulse control disorders. Ethical concerns have been raised about the use of an invasive surgical approach involving the central nervous system in patients with possible impairment in cognitive functioning and decision-making capacity. Most of the disorders in which DBS has been used might present with alterations in memory, attention, and executive functioning, which may have an impact on the mental capacity to give informed consent to neurosurgery. Depression, anxiety, and compulsivity are also common in DBS candidate disorders, and could also be associated with an impaired capacity to consent to treatment or clinical research. Despite these issues, there is limited empirical knowledge on the decision-making levels of these patients. The possible informed consent issues of DBS will be discussed by focusing on the specific treatable diseases.

## 1. Introduction

In the last two decades, a growing interest emerged for deep brain stimulation (DBS) in the treatment of movement and psychiatric disorders [1,2,3]. Despite significant advances in DBS use, surgical procedures, and outcomes, there is a substantial lack of data concerning the decision-making ability of patients undergoing DBS [4].

Different levels of evidence have highlighted the possible benefits of DBS in patients with Parkinson’s disease (PD) [5,6], essential tremor (ET) [7,8], and dystonia [9,10,11,12] who experience serious complications, or when standard pharmacological treatments were inefficacious. DBS is an accepted treatment for refractory obsessive-compulsive disorder (OCD) [13], while still limited data from research trials or case reports showed possible efficacy for treatment-resistant major depressive disorder (MDD), Tourette syndrome (TS), and impulse control disorders such as addiction, anorexia nervosa, schizophrenia, and anxiety disorders [3].

DBS received the U.S. Food and Drug Administration (FDA) approval for the treatment of patients with PD (2002) and ET (1997), as well as humanitarian device exemption for dystonia (2003) and treatment-resistant OCD (2009) [14]. The specific risk and benefit profile of DBS for each disease in which it has been used is currently studied and debated. It must be underlined that DBS for movement disorders has involved significantly more patients than psychiatric disorders, with a rate of accrual of more than 10,000 per year [1].

Both improvement and worsening of cognitive and affective symptoms, as well as impulse control disorders have been reported post-DBS [1]; however, most of the evidence indicates that DBS is beneficial particularly in movement disorders, with a minority of reports indicating the worsening of symptoms. Issues in postoperative management and rehabilitation programs also exist, and should be appropriately evaluated in the acquisition of consent to the intervention [15,16]. Postoperative management problems might also present in successfully treated patients, including those presenting a “burden of normality” syndrome, a term that refers to the possible patients’ difficulty to adapt from being chronically ill to a symptoms-free status [17]. Adaptive DBS has been recently proposed to widen the DBS therapeutic window and limit side effects; nonetheless, there is scarce evidence concerning its long-term efficacy and safety profile [18].

Candidate patients might present difficulty in properly understanding and evaluating information pertaining to the surgical procedure, including possible short and long-term consequences, as well as the experimental nature of the research in those disorders in which DBS has not yet been approved [19]. The tendency to overlook the distinction between research and ordinary treatment, a process defined as therapeutic misconception [20], is of particular importance for DBS in psychiatric disorders in view of the experimental nature of DBS in such diseases. Therapeutic misconception has been reported in patients with mood disorders undergoing DBS [21], but it has not been evaluated in other disorders, although depressive symptoms are frequently reported.

Patients with a long history of illness and characterized by poor response to previous treatments, may present frustration, alteration, of expectation in an optimistic or negative way, which may contribute in altering their mental capacity to adequately consent to treatment or clinical research [22]. Affective symptoms associated with the underlying pathology such as anxiety and mood alterations could also compromise patients’ treatment decision-making capacity [23].

In the present work, we will first synthetically discuss the informed consent doctrine with a specific focus on patients’ decision-making capacity. In the second section, we will focus on the principal disorders in which DBS has been used by synthetically reviewing existing data focusing on indications—possibly associated cognitive and affective disorders—which might impact in patients’ decision-making capacity. We will also underline the possible benefits and risks of such information being a prerequisite for the acquisition of a valid informed consent.

## 2. Informed Consent and Decision-Making Capacity

Informed consent is a prerequisite to any diagnostic, therapeutic, and clinical research procedure. To provide valid informed consent, several components are required, including: voluntariness of decision-making process, accurate and complete information disclosure, and the patient’s mental capacity to consent [24]. The capacity to give informed consent has been defined as a multidimensional construct encompassing several abilities [23].

The first of these abilities includes the patient (A) understanding the main characteristics of the disorder (diagnosis) and treatment; in the case of clinical research, this includes the nature of the project, the effects on individualized care, and the ability to withdraw. Information disclosure, in the case of DBS must include the characteristics of the surgical procedure and anaesthesia, possible risks and benefits, treatment alternatives, and predictable outcomes. A capable patient must prove able to understand and retain such information. Second, the patient must (B) appreciate the relevance and actual applicability of the previously disclosed information for the specific patient’s clinical condition. The patient must prove able to evaluate the actual presence of the disease that has been diagnosed, as well as the usefulness of the proposed treatment with associated benefits and risks. In the case of clinical research, the patient must evaluate that the objective might not result in personal benefit or even cause the possibility of reduced benefit, and that withdrawal is possible. Third, (C) a rational, logical reasoning process pertaining to the choices must be provided by the patient, including the ability to make assumptions on the possible everyday effect of treatments (or no treatment), and compare different treatment alternatives and possible outcomes. (D) Finally, a valid informed consent requires the ability to express a choice in a personal, undeleted, unambivalent way.

Several tools exist to measure patients’ decision-making capacity for treatment or clinical research [25]; the MacArthur Competence Assessment Tool for Clinical Research (MacCAT-CR) [26] and for treatment (MacCAT-T) [27] have the greatest empirical support.

Among the possible characteristics associated with the mental incapacity to consent, cognitive dysfunction has been widely acknowledged as a factor of primary importance in psychiatric and non-psychiatric samples [28,29,30,31,32]. Decisional capacity requires the intervention of multiple domains of mental functioning, such as will, inhibition, abstract reasoning, concept formation, prediction, and planning, which are strictly linked to an individual’s executive functions. Executive dysfunction has been linked to reduced or impaired treatment decision-making in psychiatric patients [31], as well as in possible DBS candidate patients [33]. Specific symptoms, such as mania and psychosis, rather than specific diagnoses, have been associated with the incapacity to give valid informed consent [34,35].

In addition to individual features, environmental factors could play a role in determining the variability of patients’ capacity to consent to treatment or clinical research, including the complexity of disclosed information, type of clinical setting, and quality of consent forms and disclosing procedures [36,37].

There is evidence supporting the frequent occurrence of cognitive impairment and psychiatric symptoms in several DBS candidate diseases such as PD [1,38,39,40], dystonia [41,42,43,44,45,46], and ET [47,48,49,50]. Thus, the risk for mental incapacity to consent should be carefully evaluated in such clinical populations undergoing DBS. The complexity of information pertaining to the DBS procedure and possible outcomes, including a margin of uncertainty concerning DBS targeting and stimulation designs, might require a significant cognitive and affective effort to be adequately appreciated and rationally manipulated.

## 3. Informed Consent Issues in DBS Candidate Diseases

### 3.1. Parkinson’s Disease

Parkinson’s disease is a chronic neurodegenerative disease, with 1% prevalence in people over 60 years [51]. There is general agreement in considering PD not just a movement disorder, but also a systemic disease characterized by numerous non-motor symptoms, such us anxiety, depression, apathy, sleep disturbances, autonomic dysfunction, and cognitive impairment (executive dysfunction, memory impairment, visuospatial dysfunction) [1]. The limits of pharmacotherapies, including the progressive loss of efficacy, dyskinesia, drug-induced psychosis, and compulsive behaviors have led an increasing use of DBS in PD. PD is also the pathology in which DBS has been most used. DBS in PD is supported by evidence from controlled trials [52]. The main targets for DBS in Parkinson’s disease are the *globus pallidus pars interna* (GPi) and the subthalamic nucleus [1]. Different targets present different risks and benefits profiles that should be acknowledged in informed consent acquisition procedures [53].

The capacity to provide valid consent to treatment in patients with PD may be altered by some frequent clinical features associated with the disease, such as cognitive impairment, mood alterations, anxiety, psychotic symptoms, and behavioral alterations (Table 1). Almost one-fourth of the patients with PD have a mild cognitive impairment at the time of diagnosis, and about 90% of patients with end-stage PD suffer from major neurocognitive disorder [38].

Alterations in executive functioning, including set shifting, planning, inhibition, and conflict resolution have been reported in patients with PD, as well as impairment in working memory, visuospatial abilities, and alterations in language [39]. The presence of specific alterations in these domains should be carefully evaluated, since it could determine an impairment of understanding and retaining of treatment-related information, evaluating possible risks and benefits, as well as of reasoning and the ability to express a choice.

DBS of the subthalamic nucleus in patients with PD is moreover associated with a progressive decrease in verbal fluency, executive functions, and working memory [54,55], implying the need for an appropriate assessment in patients who already have alterations in these domains. The presence of a major neurocognitive disorder could imply incapacity, and the consequent necessity of supported decision-making. Nonetheless, a recent pilot trial of the nucleus basalis of Meynert DBS in patients with Parkinson disease dementia enrolled six patients who have been considered capable of giving informed consent to the randomized clinical trial [56]. We believe that the level of decision-making capacity in patients with PD and major neurocognitive disorder should be carefully considered in studies on larger samples that include a specific assessment of their capacity to consent.

Depressive symptoms are one of the most frequent non-motor manifestations of PD, with 20% of cases suffering from MDD [40]; it could also alter treatment decision-making process and should be carefully evaluated. Depressive symptoms can lead to a pessimistic bias (i.e., excessive negative expectations), to an altered evaluation of subjective clinical condition and prognosis, as well as the likely consequences of the intervention, or to an alteration in reasoning and the ability to express a choice (Table 1). The prevalence of anxiety disorders in patients with PD is about 30%, and among these, the most represented disorders are generalized anxiety disorder and social phobia [57]. Anxiety symptoms can also have a negative impact on the ability to consent to treatment by interfering with understanding, appreciating, reasoning, and expressing choice abilities.

Psychotic symptoms are also frequent in patients with PD; among these, visual hallucinations and delusions are predominant, while auditory hallucinations are less frequent. Since there is much evidence linking the presence of psychotic symptoms to changes in treatment decision-making capacity in psychiatric disorders [35], the presence of psychotic symptoms in PD could be associated with potential alterations in informed consent decision-making, given their acknowledged effect in consent decision-making in psychiatric disorders.

A recent study on patients’ preferences comparing hypothetical treatment with DBS, medicine pump, or oral therapy showed that the decisions were associated with specific potential adverse effects [58]. This result is deserving of further attention, since it has possible implications also in patients’ capacity to consent/dissent to treatment.

### 3.2. Dystonia

Dystonia is a movement disorder that is characterized by sustained or intermittent muscle contractions causing abnormal, often repetitive, movements, postures, or both [59]. Patients are classified according to the clinical characteristics (age at onset, body distribution, temporal pattern, associated features) and etiology (nervous system pathology, inherited or acquired) [60]. In addition to motor symptoms, patients with idiopathic dystonia often present non-motor symptoms, including neuropsychiatric, cognitive, and sleep disorders [41,42,43,61]. There is evidence indicating that more than half of the patients affected by focal dystonia suffer from psychiatric disorders, whose onset precedes that of dystonia [42].

Depression occurs with higher rates in patients with primary focal dystonia, and in manifesting and non-manifesting DYT1 mutation carriers [41]. There is also evidence indicating an increased risk of anxiety disorders, OCD, and social phobia in dystonia [41]. Specific deficits in executive functions and executive dysfunction that are possibly linked to fronto-striatal dysfunctions have been reported in idiopathic and DYT1 dystonia [45]. Alterations in working memory, processing speed, visual motor ability, and short-term memory have also been described in adult-onset primary cranial cervical dystonia [46]. Altogether, these results deserve specific assessment of the possible impact of executive dysfunction in the capacity to consent in patients with dystonia.

Subthalamic nucleus [9] and pallidal [10] DBS proved useful in the treatment of generalized and segmental primary dystonia, while inconclusive evidence exists for focal dystonia and secondary forms [11,12]. Ventral pallidal stimulation, GPi, medullary lamina, or a combination of these have been proposed for the treatment of primary dystonia [62]. Initial data also supports long-term tolerability and the sustained effectiveness of subthalamic nucleus DBS in patients with medically refractory isolated dystonia [9]. The most common side effect of DBS in patients with dystonia is dysarthria, which is due to the unintentional diffusion of stimulation to adjacent bulbar bundles [9,63]. Among the other side effects are infections, the accidental deactivation or breakage of the electrode/internal pulse generator, wrong positioning, and lead migration [63].

Although some studies reported neuropsychological and psychiatric abnormalities in patients with dystonia who have been treated with GPi DBS, including a decline in verbal semantic fluency and increase in suicide rates, a recent neuropsychological study of a heterogeneous patient population did not confirm these risks [64]. The lack of conclusive evidence regarding these possible complications should be adequately considered in the patient information process and the acquisition of informed consent, using a precautionary viewpoint. We found no empirical studies specifically aimed at assessing the decision-making capacity of patients with dystonia undergoing DBS.

### 3.3. Essential Tremor

ET is among the most common neurological disorders; the main clinical feature is a postural or kinetic tremor, affecting the hands and forearms, although other body regions may also be involved. It has been shown that patients affected by ET may also suffer the presence of non-motor symptoms, including mood and anxiety disorders, and mild cognitive deficits [47]. MDD is frequent in patients with ET, affecting about half of the patients [48]. Depressive symptoms have been reported even more frequently, with rates of up to 79% [49]. Anxiety symptoms and social phobia have also been reported in ET [46]. Neuropsychological studies of patients with ET showed attentional and executive dysfunction, and an increased risk for mild cognitive impairment and dementia [50].

Unilateral or bilateral DBS targeted to the ventralis intermedius nucleus (VIM) of the thalamus showed significant symptom improvement in patients with ET, and it is considered a useful treatment option [7,8]. However, there is evidence indicating a possible loss of effectiveness over time of VIM thalamic DBS [65], with rates up to 70% of the treated patients [66]. This possible course should be adequately communicated and evaluated by the patient during the acquisition of consent to surgery.

Several surgical and non-surgical side effects have been reported following thalamic DBS for ET, including speech and gait problems, ataxia, balance disorders, and paresthesia [67,68]. Since unilateral VIM DBS usually presents with less adverse events, but is also associated with poorer symptoms control [56], we deem that the choice between the two procedures should be part of a shared decision-making with the patient. We found no empirical studies specifically aimed at assessing the decision-making capacity of patients with ET undergoing DBS.

### 3.4. Tourette Syndrome

TS is a neuropsychiatric disorder that is characterized by multiple, involuntary motor tics and at least one vocal tic, persisting for more than one year since the onset of the first tic (before age 18 years), according to current DSM-5 criteria [69]. Patients with TS often suffer from psychiatric comorbidities, including OCD, attention deficit hyperactivity disorder, and autism spectrum disorders [70]. A significant disease burden and impaired quality of life have been consistently reported in patients with TS, and their treatment may be complicated by a significant interindividual variability of symptoms and comorbidities [71]. Psychotherapy and pharmacological therapies are first-line treatment in patients with TS [71,72].

Data coming from the analysis of a reduced number of patients indicate that DBS is a suitable option for treatment-resistant adults with TS who are severely affected [73,74]. Nevertheless, DBS in TS is considered, to date, an experimental treatment [73]. A wide variability of potential DBS stimulus sites exists in TS [73,74], and is associated with a significant variability of effects. Several adverse effects with differences based on the DBS stimulus target have been reported; paresthesia and dysarthria [74] are the most common, followed by, gaze disturbances, visual symptoms, increased or decreased libido, mood alterations, and one case of psychosis and a suicide attempt [75]. There seems moreover to be a higher risk for post-surgical infection with DBS for TS compared with other movement disorders [75]. We found no empirical studies specifically aimed at assessing the decision-making capacity of patients with TS undergoing DBS.

### 3.5. Obsessive-Compulsive Disorder

Obsessive-compulsive disorder is characterized by the presence of obsessions, compulsions, or both, with a lifetime prevalence from 2% to 3% [76]. Untreated OCD, or treatment-refractory OCD, tend to have a chronic and disabling course [77]. First-line treatment involves integrated cognitive-behavioral psychotherapy and pharmacological therapy; however, about 10% of the patients treated do not respond to standard and second-line augmentation treatments [78].

In the last decade, DBS has been increasingly used in treatment-refractory patients, proving a potentially effective technique from a clinical and cost-efficacy perspective, with response rates up to 60% [13]. DBS in the treatment of severe treatment-resistant OCD received FDA’s humanitarian device exemption approval in 2009 [79].

In patients with OCD, the DBS has been targeted to striatal areas, including the ventral internal capsule/ventral striatum, the *nucleus accumbens*, the subthalamic nucleus, the anterior limb of the internal capsule, and the inferior thalamic peduncle [3]. Unlike the application of DBS in other disorders, the currently available data seems to suggest the absence of significant differences in efficacy among application sites [80]. Adverse effects have been described as mild, transient, and reversible; however, a meta-analysis on 31 published studies, including 116 patients, reported a significant risk of anxiety and hypomanic symptoms post-DBS [80].

The pathophysiology of OCD has long been hypothesized to be due to alterations in the orbitofrontal cortex functioning, and patients with OCD appear to have altered decision-making capacity [81]. Interestingly, anterior capsulotomy has been associated with improvement in this capacity [81]. In addition, patients with OCD tend to present higher risk-averse behavior [82]; this condition could limit the possibility of access to a potentially useful procedure that is considered abnormally risky, which could be the case for DBS.

No specific cognitive deficits nor cognitive impairment have been consistently associated with OCD, but several lines of evidence indicate a reduced performance on neuropsychological tests [83]. The possible impact of obsessions and compulsions, as well as neuropsychological features of OCD on patients’ treatment decision-making capacity deserves further assessment. We found no studies that have assessed the capacity to give consent to treatment in patients with OCD.

### 3.6. Major Depressive Disorder

MDD is the leading cause of disability among psychiatric disorders, and it has been estimated that about one-third of patients suffering from MDD do not respond to antidepressants and face a higher disease burden [84]. Some studies showed that DBS may be a viable treatment option in patients with treatment-resistant depression [85,86,87]. Nonetheless, a recent multisite, randomized, sham-controlled trial of subcallosal cingulate DBS for treatment-resistant depression showed no statistically significant antidepressant efficacy [88]. Since there is limited data coming mainly from non-controlled studies, and a sham-controlled trial proved no efficacy, DBS in treatment-resistant depression is considered an experimental treatment to be evaluated in clinical trials.

DBS in depressed patients has been targeted to the subgenual cingulate gyrus, anterior limb of internal capsule, *nucleus accumbens*, ventral capsule/ventral striatum, and superolateral branch of the medial forebrain bundle. Among the most frequent side effects were: infection, suicide, and hypomania [89]. The lack of clear evidence of efficacy, the invasive nature of the procedure, and the possible adverse events deserve specific attention when acquiring informed consent to clinical research to DBS in MDD. A study showed that patients with treatment-resistant depression who had been enrolled in studies on treatment with DBS showed a non-compromised decision-making capacity and autonomy of decision-making [90]. Despite this encouraging result, a tendency to therapeutic misconception in patients with treatment-resistant MDD has been reported [21,91], as they tended to consider the purpose of the study as specifically aimed at treating the subjects involved, rather than exploring the effectiveness of an experimental intervention.

A recent study of the impact of DBS on MDD patients’ autonomy showed that, by possibly reducing disabling symptoms such as anhedonia and fatigue, DBS could increase autonomy rather than threaten it [85]. The role of anhedonia and fatigue, their possible interaction, and their effect on patients’ decision-making ability and autonomy, considering the results in MDD, is a matter of interest in ethical empirical research that ought to be addressed also in other DBS candidate disorders in which depressive symptoms have been reported.

## 4. Conclusions

Data concerning DBS in different diseases are characterized by a heterogeneity of the levels of evidence of safety and efficacy, with more information existing for movement disorders, especially PD. Whereas in the case of psychiatric indications, there is much less robust evidence. DBS in psychiatric disorders applies only to therapeutic research; the research protocols should accordingly provide an adequate evaluation of the ability of patients to provide a valid consent to clinical research. The hypothesis of the possible ineffectiveness of DBS, which is greater in the case of research protocols, including the possibility of necessity of explanting the device, should be part of the informed consent to DBS [92].

Evaluations of the capacity to consent to treatment or clinical research could be carried out with reliable procedures and evaluation tools, considering the existence of cognitive impairments and psychiatric symptoms that could be associated with decision-making incapacity. Considering that the existence of therapeutic misconception in DBS for MDD has been demonstrated, its presence can also be hypothesized in other disorders in which experimentation is carried out, and should therefore be systematically evaluated. The possible development of post-DBS psychiatric symptoms with a likely impact on patients’ autonomy, especially hypomania, which emerged in several studies as well as alterations in executive functions and working memory, should be carefully monitored.

In conclusion, the hypothesis of a possible DBS influence on patients’ decisional autonomy deserves further empirical research. Overall, there is a significant lack of data on the ability of patients with DBS to decide whether to be treated or included in research protocols, which is an important limitation and a starting point for future studies.

## Figures and Tables

**Table 1:** Cognitive and neuropsychiatric symptoms of deep brain stimulation (DBS) candidate diseases and their possible impact on neurosurgical treatment and research decision-making capacity.

Cognitive and Neuropsychiatric Symptoms	Evidence for DBS Candidate Disease	Possible Impact on Informed Consent Decision-Making
Cognitive alterations/cognitive impairment (attention, memory, executive functions, visuospatial abilities, etc.)	Parkinson’s disease; essential tremor; dystonia; major depressive disorder	Altered understanding/retaining of treatment-related information; altered evaluation of possible risks and benefits; altered reasoning; altered ability to express a choice; therapeutic misconception. Major neurocognitive disorder/dementia might imply full incapacity
Mood alterations	Parkinson’s disease; essential tremor; dystonia; obsessive-compulsive disorder; major depressive disorder	Optimistic bias (excitement); pessimistic bias (depression); altered evaluation of his/her condition and likely consequences of the intervention; impaired reasoning; impaired expressing a choice; therapeutic misconception
Anxiety	Parkinson’s disease; essential tremor; dystonia; obsessive-compulsive disorder; Tourette syndrome; major depressive disorder	Altered evaluation of patients’ condition and likely consequences of the intervention; impaired expression of a choice; therapeutic misconception
Psychotic symptoms	Parkinson’s disease; major depressive disorder	Impaired evaluating, reasoning, expressing a choice (might include also impaired understanding in case of distracting hallucinations or pervasive delusions); therapeutic misconception
Behavioral alterations	Parkinson’s disease; dystonia, obsessive-compulsive disorder; major depressive disorder	Impaired understanding, evaluation, reasoning, expressing a choice

Note: Since DBS candidate diseases might coexist with other neuropsychiatric disorders e.g., essential tremor and mild cognitive impairment or dementia, the comorbid disease might affect the informed consent decisional capacity.
